# Tumor Testing for Somatic and Germline *BRCA1*/*BRCA2* Variants in Ovarian Cancer Patients in the Context of Strong Founder Effects

**DOI:** 10.3389/fonc.2020.01318

**Published:** 2020-07-31

**Authors:** Ana Peixoto, Pedro Pinto, Joana Guerra, Manuela Pinheiro, Catarina Santos, Carla Pinto, Rui Santos, Carla Escudeiro, Carla Bartosch, Rita Canário, Ana Barbosa, Alfredo Gouveia, Almerinda Petiz, Miguel Henriques Abreu, Susana Sousa, Deolinda Pereira, João Silva, Manuel R. Teixeira

**Affiliations:** ^1^Department of Genetics, Portuguese Oncology Institute of Porto (IPO Porto), Porto, Portugal; ^2^Cancer Genetics Group, IPO Porto Research Center (CI-IPOP), Portuguese Oncology Institute of Porto (IPO Porto), Porto, Portugal; ^3^Department of Pathology, Portuguese Oncology Institute of Porto (IPO Porto), Porto, Portugal; ^4^Cancer Biology and Epigenetics Group, CI-IPOP, IPO Porto, Porto, Portugal; ^5^Epithelial Interactions in Cancer Lab, Instituto de Investigação e Inovação em Saúde (I3S)/Instituto de Patologia e Imunologia Molecular da Universidade Do Porto (IPATIMUP), University of Porto, Porto, Portugal; ^6^Graduate Program in Areas of Basic and Applied Biology, Instituto de Ciências Biomédicas Abel Salazar (ICBAS), University of Porto, Porto, Portugal; ^7^Department of Gynecology, Portuguese Oncology Institute of Porto (IPO Porto), Porto, Portugal; ^8^Department of Medical Oncology, Portuguese Oncology Institute of Porto (IPO Porto), Porto, Portugal; ^9^Institute of Biomedical Sciences Abel Salazar (ICBAS), University of Porto, Porto, Portugal

**Keywords:** *BRCA1/BRCA2*, ovarian cancer, PARPi, NGS, founder variants, tumor testing

## Abstract

Deleterious variants in the *BRCA1/BRCA2* genes and homologous recombination deficiency (HRD) status are considered strong predictors of response to poly (ADP-ribose) polymerase (PARP) inhibitors (PARPi). The introduction of PARPi in clinical practice for the treatment of patients with advanced ovarian cancer imposed changes in the molecular diagnosis of *BRCA1*/*BRCA2* variants. *BRCA1*/*BRCA2* tumor testing by next-generation sequencing (NGS) can detect simultaneously both somatic and germline variants, allowing the identification of more patients with higher likelihood of benefiting from PARPi. Our main goal was to determine the frequency of somatic and germline *BRCA1*/*BRCA2* variants in a series of non-mucinous OC, and to define the best strategy to be implemented in a routine diagnostic setting for the screening of germline/somatic variants in these genes, including the *BRCA2* c.156_157insAlu Portuguese founder variant. We observed a frequency of 19.3% of deleterious variants, 13.3% germline, and 5.9% somatic. A higher prevalence of pathogenic variants was observed in patients diagnosed with high-grade serous ovarian cancer (23.2%). Considering the frequencies of the c.3331_3334del and the c.2037delinsCC *BRCA1* variants observed in this study (73% of all *BRCA1* pathogenic germline variants identified) and the limitations of NGS to detect the *BRCA2* c.156_157insAlu variant, it might be cost-effective to test for these founder variants with a specific test prior to tumor screening of the entire coding regions of *BRCA1* and *BRCA2* by NGS in patients of Portuguese ancestry.

## Introduction

Pathogenic germline variants in the breast cancer susceptibility genes *BRCA1* and *BRCA2* increase the risk for the development of ovarian cancer (OC) in carriers. The cumulative OC risk at age 80 years is 44 and 17% for *BRCA1* and *BRCA2* variant carriers, respectively (1). Women unselected for family history present germline *BRCA1*/*BRCA2* variants in 14% of the cases when having any epithelial OC and in ~17% of the cases with a high-grade serous ovarian cancer (HGSOC) diagnosis (2, 3). Furthermore, somatic mutations were observed in these genes in an additional 3% of HGSOC (2). In total, up to 50% of HGSOC have homologous recombination defects related with loss of function of BRCA1 or BRCA2 or other homologous recombination (HR) pathway proteins (2).

BRCA1 and BRCA2 are critical proteins in the process of HR repair of double-strand DNA breaks (DSBs). BRCA1/BRCA2-deficient cancers are recognized as the main responders to a class of drugs known as poly (ADP-ribose) polymerase (PARP) inhibitors (PARPi) (4, 5). PARPi blocks the base excision repair (BER) pathway, which is involved in the repair of DNA single*-*strand breaks, leading to the formation of DSBs that cannot be accurately repaired in HR-deficient cells and consequently to cell death. (4, 6). Deleterious variants in the *BRCA1*/*BRCA2* genes and homologous recombination deficiency (HRD) status are strong predictors of response to PARPi (7). The PARPi olaparib (Lynparza) was the first-in-class agent to gain approval for treatment in OC by the European Medicines Agency (EMA) for use as maintenance therapy of patients with platinum-sensitive relapsed, BRCA-mutated advanced epithelial ovarian, fallopian tube or primary peritoneal cancer and by the U.S. Food and Drug Administration (FDA) as monotherapy for patients with germline BRCA mutations, who have received three or more prior lines of chemotherapy (8). Consequently, it became mandatory to determine the *BRCA1*/*BRCA2* mutational status to be able to select HGSOC patients for PARPi therapy. At that time, however, the regulatory approvals of FDA and EMA differed, as the latter also considered HGSOC patients with somatic *BRCA1/BRCA2* mutations as eligible for PARPi therapy. After that, FDA and EMA approved olaparib for the maintenance treatment in the recurrent setting, regardless of BRCA status, and, more recently, in patients with newly diagnosed BRCA-mutated advanced OC. Therefore, molecular diagnosis algorithms in OC patients had to be updated, not only because of the availability of the new therapy for HGSOC, but also because molecular diagnostic labs would have to consider the detection of somatic *BRCA1/BRCA2* mutations. Currently, there is no consensus regarding in which order one should undertake germline and tumor *BRCA1/BRCA2* testing in HGSOC patients, but it is generally recommended to perform both (9, 10). Although the tumor testing strategy would need subsequent test in a blood sample of specific variants to evaluate if they are of germline or somatic origin, this would be more cost effective than performing full tumor testing after a negative full germline test to identify the rarer somatic variants. Since *BRCA1*/*BRCA2* tumor testing can detect simultaneously both somatic and germline variants, with the exception of some variants like rearrangements, a higher number of patients who may benefit from PARPi can be identified at a faster turnaround time and at a lower cost (9).

In this study, we aimed to estimate the prevalence of germline and somatic *BRCA1* and *BRCA2* variants in a consecutive series of non-mucinous ovarian cancer patients and to evaluate the advantages and limitations of the tumor testing first strategy.

## Materials and Methods

### Patient Samples

A consecutive series of patients with non-mucinous OC treated at the Portuguese Oncology Institute of Porto from January 2016 to December 2017 (135 patients), from whom formalin–fixed and paraffin–embedded (FFPE) tissue and a peripheral blood sample were available, were analyzed. All patients included in the study were referred for genetic counseling and written informed consent was obtained together with collection of cancer family history and subsequent calculation of the Manchester Score, which estimates the probability of finding a germline *BRCA1*/*BRCA2* variant (11). Tumor samples from 10 patients with known pathogenic germline variants in *BRCA1*/*BRCA2* were collected as validation controls. FFPE samples were obtained, with hematoxylin and eosin-stained slides carefully reviewed by an experienced pathologist in gynecological tumors, who delimited areas with >50% tumor cells. DNA extraction was performed from tumor tissue using the *cobas*® DNA Sample Preparation Kit (Roche Diagnostics, Basel, Switzerland) according to the manufacturer's protocol and DNA quality was evaluated using the Qubit® 2.0 Fluorometer with the Qubit dsDNA HS Assay Kit (Thermo Fisher Scientific, Waltham, MA, USA). DNA was extracted from peripheral blood leucocytes using a standard protocol. Blood samples were used to confirm whether the variants found in the tumor samples were germline or somatic, to search for the *BRCA1/BRCA2* germline founder variants and to test for large genomic rearrangements (LGRs), the latter in patients with a Manchester score equal or higher than 15.

One hundred and nine cases (80.7%) had tumors with a pure serous histology, including 95 (70.4%) HGSOC and 14 (10.4%) LGSOC. Twenty-one cases (15.6%) were of non-serous histology, including 10 (7.4%) clear cell, nine (6.7%) endometrioid, and two (1.5%) mixed with clear cell, and endometrioid histology. There were also four (3%) carcinosarcomas, and one (0.7%) mixed carcinoma with clear cell and HGSOC components. Ninety-one FFPE samples (67.4%) were obtained prior to treatment, 27 (20%) post treatment with chemotherapy, and for 17 samples (12.6%) it was not possible to obtain that information.

### Next-Generation Sequencing

Next-generation sequencing (NGS) was performed in all FFPE tumor samples using the BRCA Tumor MASTR™ Plus Dx (Multiplicom, Niel, Belgium), an amplicon based NGS kit targeting the full coding sequence and adjacent intronic regions of the *BRCA1*/*BRCA2* genes, following the manufacturer's protocol. Sequencing was carried out using a standard flow cell in the MiSeq platform (Illumina, Inc., San Diego, CA, USA) in 2 × 250 bp paired-end runs. Sequencing and bioinformatic analysis was carried out as previously described (12). All deleterious variants and variants of uncertain significance (VUS) identified by NGS were confirmed by Sanger sequencing following a standard protocol.

### Large Genomic Rearrangements and Founder Variants Screening

The detection of *BRCA1/BRCA2* LGRs and Portuguese founder variants was performed in DNA extracted from peripheral blood samples. Multiplex Ligation-dependent Probe Amplification (MLPA; MRC-Holland, Amsterdam, Netherlands) was used to detect *BRCA1*/*BRCA2* LGRs, according to the manufacturer's instructions. Screening of the *BRCA2* c.156_157insAlu variant was performed in all patients according to the protocol previously described by us (13). Screening of the *BRCA1* c.2037delinsCC and c.3331_3334del variants was performed using KASPar SNP genotyping technology (LGC, Teddington, UK) on a Roche LightCycler 480 Real-Time PCR System, according to manufacturer's instructions. KASPar assay primers were designed using the Primer-BLAST design tool (14) and are available upon request. Genotyping results were analyzed using the LightCycler 480 Software 1.5.0. Positive samples were confirmed by Sanger sequencing following a standard protocol.

### Loss of Heterozygosity (LOH)

VAF was used to infer biallelic inactivation by deletion of the second allele. LOH presence was evaluated in patients with *BRCA1* or *BRCA2* germline pathogenic variants and VUS that were called in a heterozygous state in the tumor samples. LOH was considered present when the germline *BRCA1/BRCA2* VAF was >60%, and/or at least two informative (heterozygous) single nucleotide variants (SNVs) showed a VAF <0.4 or >0.6 (15).

## Results

### Variant Detection

A total of 10 FFPE tumor DNA samples from OC patients with known pathogenic germline variants were used to validate the NGS assay, including the bioinformatic analysis. This sample set included deletions, duplications, point mutations, and the *BRCA2* c.156_157insAlu Portuguese founder variant (Table 1), which is not detectable using standard sequencing methodologies in FFPE samples (12). Regarding the known germline point mutations, the concordance between Sanger sequencing in peripheral blood samples and the NGS-based tumor test on FFPE samples was 100% (8/8). As expected, the germline *BRCA2* c.156_157insAlu variant was not called by the NGS tumor assay pipeline described above.

**Table 1:** Known pathogenic germline variants used to validate the NGS assay.

**Gene**	**HGVS coding**	**HGVS protein**	**Tumor**	**Blood**	**RD tumor**	**VAF tumor %**
*BRCA1*	c.3331_3334del	p.(Gln1111AsnfsTer5)	Positive	Positive	2,812	62
*BRCA1*	c.2490_2497dup	p.(Leu833CysfsTer16)	Positive	Positive	457	86
*BRCA1*	c.2086dup	p.(Thr696AsnfsTer16)	Positive	Positive	1,247	70
*BRCA1*	c.5278-1G>T		Positive	Positive	840	71
*BRCA1*	c.3331_3334del	p.(Gln1111AsnfsTer5)	Positive	Positive	534	80
*BRCA1*	c.470_471del	p.(Ser157Ter)	Positive	Positive	3,729	80
*BRCA1*	c.3817C>T	p.(Gln1273Ter)	Positive	Positive	2,275	68
*BRCA1*	c.2906del	p.(Asn969IlefsTer31)	Positive	Positive	5,917	85
*BRCA2*	c.156_157insAlu		Negative	Positive		
*BRCA2*	c.156_157insAlu		Negative	Positive		

*RD, read depth; VAF, variant allele frequency*.

The NGS-based tumor test was performed in 136 ovarian tumor samples derived from 135 patients. We detected 27 pathogenic variants in 26 patients (19.3%; Table 2): 16 patients with a deleterious *BRCA1* variant (61.5%) and 10 patients with a deleterious *BRCA2* variant (38.5%). A total of 18 (13.3%) patients had germline variants (11 in the *BRCA1* gene and seven in the *BRCA2* gene) and eight (5.9%), including one patient with two pathogenic variants in the *BRCA1* gene, presented mutations that were found to be somatic (five in *BRCA1* and three in *BRCA2)*. The most frequent deleterious variant was the *BRCA1* c.3331_3334del, detected in 4.4% (6/135) of the tumors and representing 22.2% of the pathogenic variants found in this series. This variant together with c.2037delinsCC represents 73% (8/11) of all the *BRCA1* pathogenic germline variants identified.

**Table 2:** Pathogenic variants identified.

**Patient**	**Histological type**	**Gene**	**HGVS coding**	**HGVS protein**	**Tumor**	**Blood**	**RD tumor**	**VAF tumor %**	**MS**
1	HGSOC	*BRCA1*	c.1192_1193del	p.(Ser398ThrfsTer2)	Positive	Negative	5,885	52	15
2	Endometrioid	*BRCA1*	c.1459_1463delinsTAT	p.(Val487TyrfsTer2)	Positive	Negative	13,929	19	12
3	HGSOC	*BRCA1*	c.1058G>A	p.(Trp353Ter)	Positive	Positive	5,170	55	15
4	HGSOC	*BRCA1*	c.2037delinsCC	p.(Lys679AsnfsTer4)	Positive	Positive	12,302	63	28
5	HGSOC	*BRCA1*	c.2037delinsCC	p.(Lys679AsnfsTer4)	Positive	Positive	6,419	77	18
6	HGSOC	*BRCA1*	c.3331_3334del	p.(Gln1111AsnfsTer5)	Positive	Positive	522	87	47
7	HGSOC	*BRCA1*	c.3331_3334del	p.(Gln1111AsnfsTer5)	Positive	Positive	6,015	92	18
8	HGSOC	*BRCA1*	c.3331_3334del	p.(Gln1111AsnfsTer5)	Positive	Positive	6,118	86	28
9	HGSOC	*BRCA1*	c.3331_3334del	p.(Gln1111AsnfsTer5)	Positive	Positive	7,995	87	17
10	HGSOC	*BRCA1*	c.3331_3334del	p.(Gln1111AsnfsTer5)	Positive	Positive	3,544	84	31
11	HGSOC	*BRCA1*	c.3331_3334del	p.(Gln1111AsnfsTer5)	Positive	Positive	4,700	71	19
12	Carcinosarcoma	*BRCA1*	c.211A>G	p.(Arg71Gly)	Positive	Positive	2,048	84	22
13	Carcinosarcoma	*BRCA1*	c.1016dup	p.(Val340GlyfsTer6)	Positive	Negative	3,887	57	15
14	HGSOC	*BRCA1*	c.3817C>T	p.(Gln1273Ter)	Positive	Positive	5,687	91	41
15	HGSOC	*BRCA1*	c.4411_4412del	p.(Gly1471ProfsTer4)	Positive	Negative	8,921	25	15
15	HGSOC	*BRCA1*	c.4485-2A>C		Positive	Negative	3,610	6	
25	HGSOC	*BRCA1*	c.116G>T	p.(Cys39Phe)	Positive	Negative	30,150	24	18
16	HGSOC	*BRCA2*	c.8488-1G>A		Positive	Positive	2,838	49	23
17	HGSOC	*BRCA2*	c.5073dup	p.(Trp1692MetfsTer3)	Positive	Positive	6,900	78	23
18	HGSOC	*BRCA2*	c.4964dup	p.(Tyr1655Ter)	Positive	Positive	3,592	80	12
19	HGSOC	*BRCA2*	c.5073dup	p.(Trp1692MetfsTer3)	Positive	Positive	9,183	78	19
20	HGSOC	*BRCA2*	c.4964dup	p.(Tyr1655Ter)	Positive	Positive	2,708	70	20
21	Endometrioid	*BRCA2*	c.5436del	p.(Glu1812AspfsTer3)	Positive	Negative	5,638	69	19
22	HGSOC	*BRCA2*	c.5950_5961delinsTGCT	p.(Lys1984CysfsTer16)	Positive	Negative	21,046	65	15
23	HGSOC	*BRCA2*	c.9379_9400del	p.(Trp3127AlafsTer29)	Positive	Negative	1,357	6	18
24	HGSOC	*BRCA2*	c.156_157insAlu		Negative	Positive			21
34	HGSOC	*BRCA2*	c.7975A>G	p.(Arg2659Gly)	Positive	Positive	873	56	18

*HGSOC, high-grade serous ovarian cancer; RD, read depth; VAF, variant allele frequency; MS, Manchester score*.

We also detected 12 VUS in 11 patients (8.1%). Within this group, eight (5.9%) patients had a germline VUS and four (3%) patients had a somatic VUS (Table 3). One patient had one VUS in each of the genes, one somatic *BRCA1* VUS and one germline *BRCA2* VUS.

**Table 3:** Variants of unknown significance identified.

**Patient**	**Histological type**	**Gene**	**HGVS coding**	**HGVS protein**	**Tumor**	**Blood**	**VAF tumor %**	**MS**
26	HGSOC	*BRCA1*	c.898G>A	p.(Glu300Lys)	Positive	Negative	19	12
27	HGSOC	*BRCA1*	c.994C>T	p.(Arg332Trp)	Positive	Positive	19	15
29	HGSOC	*BRCA1*	c.5420T>G	p.(Ile1807Ser)	Positive	Negative	38	13
30	Clear cell	*BRCA2*	c.19G>C	p.(Glu7Gln)	Positive	Negative	15	10
31	HGSOC	*BRCA2*	c.1343G>A	p.(Arg448His)	Positive	Positive	49	21
32	HGSOC	*BRCA2*	c.3256A>T	p.(Ile1086Leu)	Positive	Positive	64	15
33	HGSOC	*BRCA2*	c.4933_4935del	p.(Lys1645del)	Positive	Positive	45	12
23	HGSOC	*BRCA2*	c.6351_6377del	p.(Val2118_Cys2126del)	Positive	Negative	69	
26	HGSOC	*BRCA2*	c.7435+6G>A		Positive	Positive	56	
35	HGSOC	*BRCA2*	c.8036A>G	p.(Asp2679Gly)	Positive	Positive	50	13
36	LGSOC	*BRCA2*	c.8902A>G	p.(Thr2968Ala)	Positive	Positive	50	14
37	HGSOC	*BRCA2*	c.9364G>C	p.(Ala3122Pro)	Positive	Positive	85	27

*HGSOC, high-grade serous ovarian cancer; LGSOC, low grade serous ovarian cancer; VAF, variant allele frequency; MS, Manchester score*.

In one of the samples, no deleterious variant was identified using the variant filters previously described. However, when reviewing the data, a pathogenic variant (*BRCA1* c.1459_1463delinsTAT) with a 4% VAF was identified that had been discarded by the software due to low VAF (<5%). This tumor sample was obtained post neoadjuvant chemotherapy (paclitaxel and carboplatin) and another available sample, prior to treatment, was subsequently analyzed. The same *BRCA1* pathogenic mutation was detected in the second analysis, but now with a 19% VAF.

### LOH

LOH was evaluated in patients with *BRCA1* or *BRCA2* germline pathogenic variants (*n* = 11 for *BRCA1*; *n* = 6 for *BRCA2*) and VUS (*n* = 1 for *BRCA1*; *n* = 7 for *BRCA2*) in the tumor samples. In the sample with *BRCA2* c.156_157insAlu, LOH was not possible to evaluate since this variant was not called by the software and there were no informative SNVs. The subset of germline pathogenic variants had a mean VAF of 80% and 69% for *BRCA1* and *BRCA2* genes, respectively. The subset of germline VUS had a mean VAF of 53%. We considered that LOH occurred in 10 out of 11 patients (91%) and in 4 out of 6 patients (67%) with a *BRCA1* and *BRCA2* germline pathogenic variant, respectively. Loss of the wild type allele was not observed in the tumor from the patient with a germline *BRCA1* VUS. In patients with germline *BRCA2* VUS, loss of the wild type allele was seen in 29% (2/7) of the tumor samples.

### Manchester Score

The Manchester score was calculated for 133 patients (three patients belonged to the same family) and a median score of 15 was obtained. The median score was 14 for patients where no germline pathogenic variants or VUS were identified (*n* = 107), 15 for patients with a germline VUS (*n* = 8, Table 3), and 21 for patients with a germline pathogenic variant (*n* = 18, Table 2).

### Frequency of Mutations by Histology

A higher prevalence of pathogenic variants was observed in patients diagnosed with HGSOC, namely 17 of 95 (17.9%) patients with germline variants and 22 of 95 (23.2%) patients with germline/somatic variants. Four additional tumors, out of the 40 with other histologies (10%), had a deleterious germline or somatic BRCA mutation, namely 2 of 9 (22.2%) endometrioid carcinomas, both of which were high grade, and 2 of 4 (50%) carcinosarcomas.

## Discussion

The National Authority of Medicines and Health Products (Infarmed) approved olaparib in Portugal as maintenance therapy only in HGSOC patients with a germline or somatic BRCA mutation. Therefore, it became important to evaluate whether a tumor-testing-first strategy would be the most cost-effective option, allowing for the simultaneous detection of both germline and somatic *BRCA1*/*BRCA2* variants. However, the detection of somatic mutations depends on DNA extraction from FFPE tumor material, which is usually of poor quality and highly fragmented. Additionally, tumor samples are very heterogeneous and contamination with DNA from normal tissue is often an issue. In order to detect somatic mutations in addition to the germline variants, it is necessary to use a methodology with high sensitivity and specificity, such as the use of NGS after tumor macrodissection of the tumor areas marked by a pathologist. However, accurate detection of LGRs in tumor samples with NGS is not straightforward. Moreover, the specific variant c.156_157insAlu represents about 50% of the *BRCA2* pathogenic variants identified in the Portuguese population, but it is not detected by standard sequencing technologies neither by common bioinformatic approaches using NGS data (12). In this study, we used Multiplicom BRCA MASTR Dx assay for the detection of *BRCA1*/*BRCA2* variants using DNA extracted from FFPE tumor samples, for which it has CE-IVD marking. Furthermore, our bioinformatic analysis used Sophia Genetics software which also obtained CE-IVD marking. Our main goal was to determine the frequency of somatic and germline *BRCA1*/*BRCA2* variants in a series of non-mucinous OC, and to define the best strategy to be implemented in a routine diagnostic setting for screening of germline/somatic variants in these genes, including the *BRCA2* c.156_157insAlu founder variant.

The first task of this work consisted in the analysis of FFPE tumor DNA samples from OC patients with known pathogenic germline variants to validate the NGS assay. All germline point mutations were detected by the NGS-based tumor test, allowing us to implement this technique in a routine diagnostic setting. However, as expected, the germline *BRCA2* c.156_157insAlu variant was not called by the NGS tumor assay pipeline, using the software Sophia DDM®, in two tumor samples from the validation series. Taking this into account, blood samples from the 135 patients were analyzed to search for the *BRCA2* c.156_157insAlu germline variant. In this study, we detected the presence of germline pathogenic variants in 13.3% of the 135 patients studied, which is comparable to previous studies. The frequency of *BRCA1* and *BRCA2* germline variants in women with ovarian cancer varies in the literature (6–41%), with the lowest prevalence observed in unselected series of patients with OC (16–18). A higher prevalence of *BRCA1/BRCA2* variants (>15%) has been consistently described in patients with HGSOC (16, 19). Although we observed a predominance (23.2%) of *BRCA1*/*BRCA2* variants in patients with HGSOC, these alterations were not exclusively associated with this group, as they were also frequently found in carcinomas with other histologies (10%). These findings corroborate those obtained by Pennington et al. (20), which found HR gene variants (germline and somatic) to be also common in carcinomas with non-HGSOC histologies. In this work, we identified a *BRCA1/BRCA2* deleterious variant frequency of 50% (2/4) in ovarian carcinosarcomas. Although this frequency can be overestimated due to the limitation of a small sample size, the association of ovarian carcinosarcomas with *BRCA1/BRCA2* pathogenic variants has already been described in the literature (20–22), including two (17%) out of 12 ovarian carcinosarcomas, one with a germline and the other with a somatic mutation. Indeed, there are a few studies indicating that ovarian carcinosarcomas and HGSOC may arise from the same precursor lesion in the Fallopian tube (serous intraepithelial carcinoma) (23).

**Figure 1:**
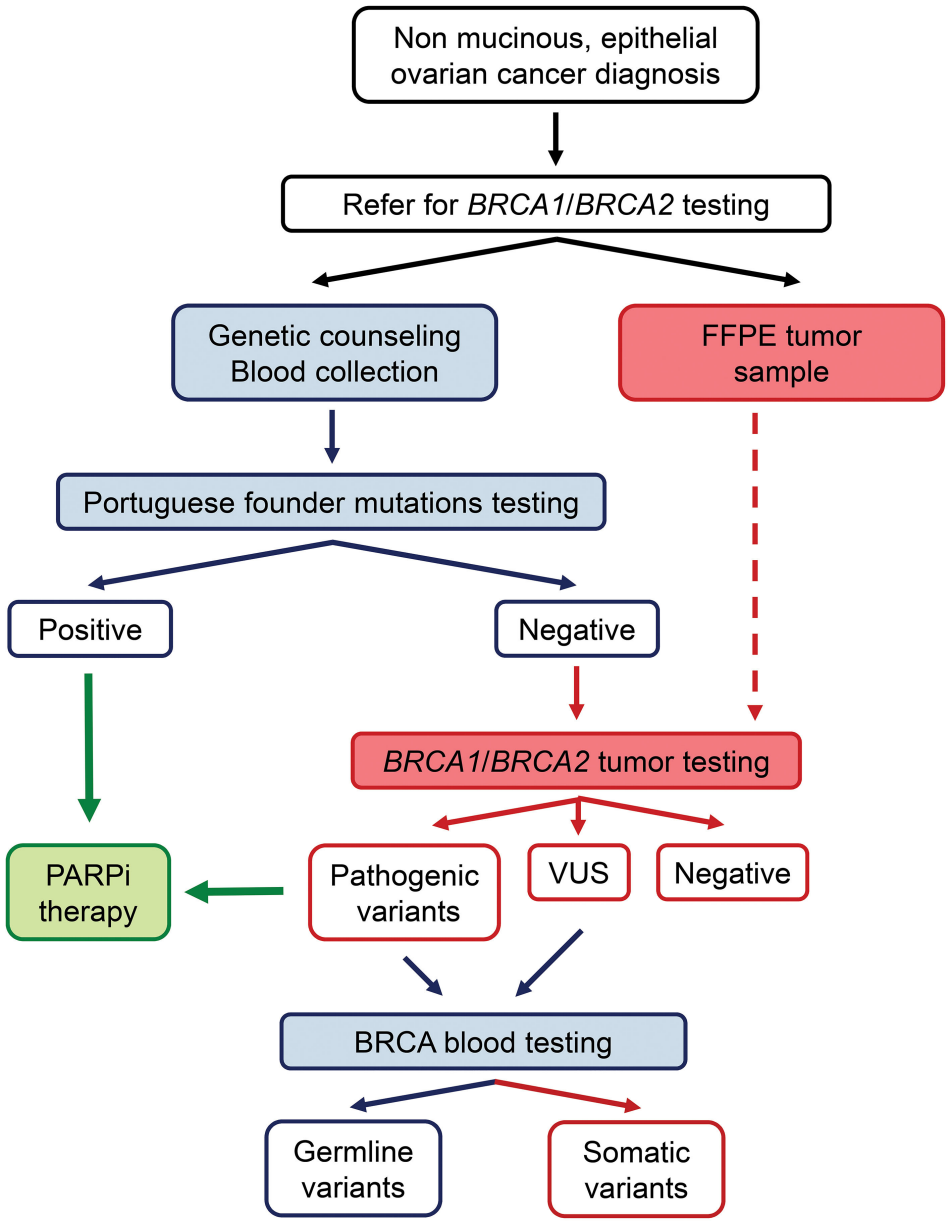
Strategy for detection of germline and somatic *BRCA1*/*BRCA2* mutations in ovarian cancer patients: A specific blood test for the detection of the three most common mutations in our population is performed before tumor screening of the entire coding regions of *BRCA1* and *BRCA2* by NGS. Blood samples from these patients are used to confirm whether the variants found in the tumor samples are germline or somatic. Patients with pathogenic variants in *BRCA1*/*BRCA2* genes are eligible for PARPi therapy.

The identification of specific and recurrent/founder variants in any given population allows a more efficient and cost-saving mutational screening approach. In our previous work, we demonstrated that two variants in *BRCA1* (c.2037delinsCC and c.3331_3334del) and one in *BRCA2* (c.156_157insAlu) together represent about 50% of all deleterious variants found in Portuguese hereditary breast and ovarian cancer families mostly originated from northern Portugal (13). These data allowed us to define our current strategy of starting the genetic study of all families by testing these variants before the screening of the entire coding regions of the *BRCA1* and *BRCA2* genes. In this study, we identified six patients with the *BRCA1* c.3331_3334del (4.4%), two with the *BRCA1* c.2037delinsC variant (1.5%), and one patient with the *BRCA2* c.156_157insAlu variant (0.7%). Together, these three variants represent 50% (9/18) of all germline deleterious variants found in this series, indicating that it might be cost-effective to test for these founder variants with a specific test prior to tumor screening of the entire coding regions of *BRCA1* and *BRCA2* by NGS in patients of Portuguese ancestry (Figure 1). Furthermore, since the detection of LGRs by NGS in FFPE samples, including the *BRCA2* c.156_157insAlu variant, is not yet optimized due to the low quality of FFPE samples and the possibility of somatic chromosomal deletions and gains that might shield germline LGRs, blood samples from these patients must be collected to test for this specific variant and for germline LGRs [which are relatively rare in our population (13)] at least in patients with a Manchester score higher than 14. Nevertheless, any strategy for the detection of *BRCA1* and *BRCA2* variants must be adapted to specific populations, considering the presence and nature of recurrent and/or founder variants and the ability of current methodologies to detect them in FFPE tissue. In the future, it might be time-saving to optimize the bioinformatics pipeline to detect all variant types in FFPE tissue, making the blood sample only necessary to determine the eventual germline origin of a variant identified in the initial tumor testing by NGS (eventually preceded by founder variant testing in the tumor if considered cost-effective).

In this study, Manchester score was determined for 133 patients. The median of this score was higher for patients with pathogenic germline variants in comparison to patients where no germline variants or a VUS was identified, reflecting that a family history of breast and/or ovarian cancer increases significantly the chance of identifying women with a germline *BRCA1*/*BRCA2* variant. In general, a 10% estimated probability of finding a germline *BRCA1*/*BRCA2* variant is considered to be cost-effective for DNA testing (24). Although a strong family history increases the chance of identifying these variants, it has been reported that family history may be absent in a significant percentage of germline *BRCA1*/*BRCA2* variant carriers (25). Recently, an overall probability of a germline *BRCA1*/*BRCA2* variant above 10% was described for all women with epithelial OC (26). Therefore, it is recommended to refer all women with these tumors for genetic risk evaluation and DNA analysis. In the present series, however, only 1 out of 18 (6%) patients with a *BRCA1*/*BRCA2* pathogenic germline variant had a prior probability lower than 10%. Although BRCA variant testing for ovarian cancer patients must be done in the context of targeted therapy to estimate the potential clinical benefit, in our population the majority of patients with a germline BRCA variant would have been identified based on personal and family history of breast and/or ovarian cancer, revealing that the current model for genetic testing based in risk assessment using familial risk models is still an accurate tool to select patients for germline genetic testing in our population.

It is still not entirely clear if the magnitude of benefit from PARPi for a patient with OC harboring *BRCA1*/*BRCA2* somatic mutations is the same as for those with a germline variant (27). Phase 3 trials that included germline and somatic BRCA mutated patients revealed similar outcomes between these two groups (28, 29). Somatic mutations were ascertained in several studies, with report rates varying from 4 to 7% (20, 30). Our study revealed the presence of somatic *BRCA1*/*BRCA2* pathogenic mutations in 5.9% of the 135 patients studied and in 30.8% of all the patients with pathogenic variants, which is comparable to previous studies. Testing both tumor and blood samples increased the proportion of pathogenic variants identified in OC patients from 13.3 to 19.3% (17.9 to 23.2% in patients with HGSOC), allowing the identification of more patients with higher likelihood of benefiting from PARPi.

Several factors can influence the number of variants detected in a tumor. A factor that must be taken into account is the quality of FFPE samples for DNA analysis (9). For instance, the age of the FFPE block has a significant impact on the quality and the number of variants detected (31). Despite the limited number of studies evaluating the mutation profile in pre- and post-chemotherapy OC specimens, tumor mutational shifts have been described after chemotherapy (32). This phenomenon may be due to pre-existing intra-tumoral heterogeneity and sampling bias, cytotoxic therapy applying selective pressure, or direct drug-induced genetic aberrations (32). One patient from our series was tested in two different samples, one obtained prior to and the other after treatment with chemotherapy. No mutations were detected in the post-treatment sample using the cutoff of a minimum 5% VAF, although a *BRCA1* pathogenic mutation was present with a 4% VAF. In the sample that was obtained prior to treatment, the same *BRCA1* pathogenic variant was present with a VAF of 19%. This finding highlights the importance of selecting the most suitable sample for *BRCA1*/*BRCA2* tumor testing in OC patients. Although the analysis of metastatic tissue at the time of progression may provide a more accurate indication of tumors likely to respond to PARPi treatment, the information available from clinical trials relates to the analysis of primary ovarian tumors (9). While there are no recommendations about the timing of *BRCA1/BRCA2* mutation analysis concerning pre- and post-therapeutic tumor samples, an adequate collection of tumor samples with a high tumor content prior to surgery is advised (9).

It is accepted that tumors with *BRCA1*/*BRCA2* germline pathogenic variants usually exhibit LOH, resulting from deletion of the wild type allele, which can be inferred from the high VAF of the mutant allele. BRCA locus-specific LOH in germline *BRCA1*/*BRCA2* carriers has been associated with sensitivity to DNA damaging agents. In a recent work, absence of LOH was observed in 7% of *BRCA1* and 16% of *BRCA2* ovarian tumors and was correlated with decreased overall survival in ovarian cancers treated with platinum chemotherapy (33). In this study, LOH was observed in 91 and 67% of ovarian tumors with a *BRCA1* and *BRCA2* germline pathogenic mutation, respectively, which is in accordance with previous reports. Given that most ovarian tumors with germline BRCA deleterious variants show LOH and loss of the wildtype allele in tumor tissue provides strong evidence for a deleterious germline mutation, we can use LOH status to provide some evidence about the clinical significance of VUS in ovarian cancer tumors. Whereas, LOH was observed in more than 80% of the patients with *BRCA1*/*BRCA2* germline pathogenic variants, loss of the wild type allele was observed in about 25% of the patients with *BRCA1*/*BRCA2* VUS. These results suggest that most of the germline VUS identified are probably not pathogenic. One of these variants is the c.994C>T in the *BRCA1* gene, which is described in ClinVar (ID 55775) as a VUS and was detected in the tumor sample with a VAF of 19%, which is relatively low for a germline variant. On the other hand, the *BRCA2* c.9364G>C variant was identified with a VAF of 85%, which is suggestive of LOH and pathogenicity. Nevertheless, LOH might be the result of genomic instability, therefore, additional studies will be required to further characterize these variants.

In conclusion, we have characterized the mutation spectrum of *BRCA1/BRCA2* in a consecutive series of ovarian carcinomas, observing a frequency of 19.3% of deleterious variants, 13.3% germline, and 5.9% somatic. Considering the frequencies of the variants observed in our study and the limitations of NGS, we recommend performing a specific blood test for the detection of the three most common variants in our population prior to tumor screening of the entire coding regions of *BRCA1* and *BRCA2* by NGS. Any deleterious variant identified in the tumor testing, which by itself is predictive of better response to PARPi, should subsequently be evaluated for its germline or somatic origin.

## Data Availability Statement

The original contributions presented in the study are publicly available. This data can be found here: European Nucleotide Archive (ENA) with accession number PRJEB38270 (https://www.ebi.ac.uk/ena/data/view/PRJEB38270).

## Ethics Statement

Ethical review and approval was not required for the study on human participants in accordance with the local legislation and institutional requirements. The patients/participants provided their written informed consent to participate in this study.

## Author Contributions

APei, PP, and JG performed experiments. APei, PP, JG, MP, CS, CP, RS, and CE analyzed data. CB, RC, AB, AG, APet, MA, SS, DP, and JS provided samples and data. APei, PP, and MT wrote the manuscript. MT designed and supervised the study. All authors critically revised and approved the manuscript.

## Conflict of Interest

The authors declare that the research was conducted in the absence of any commercial or financial relationships that could be construed as a potential conflict of interest.
